# Skull base reconstruction with nasoseptal flap in oncologic cases: can narrow-band imaging help? How we do it

**DOI:** 10.1308/rcsann.2025.0017

**Published:** 2025-04-24

**Authors:** M Stavrakas, Y Kanaan, R Al-Ashqar, H Khalil, S Muquit

**Affiliations:** ^1^ University Hospitals Plymouth NHS Trust, UK; ^2^ University of Plymouth, UK

**Figure 1:**
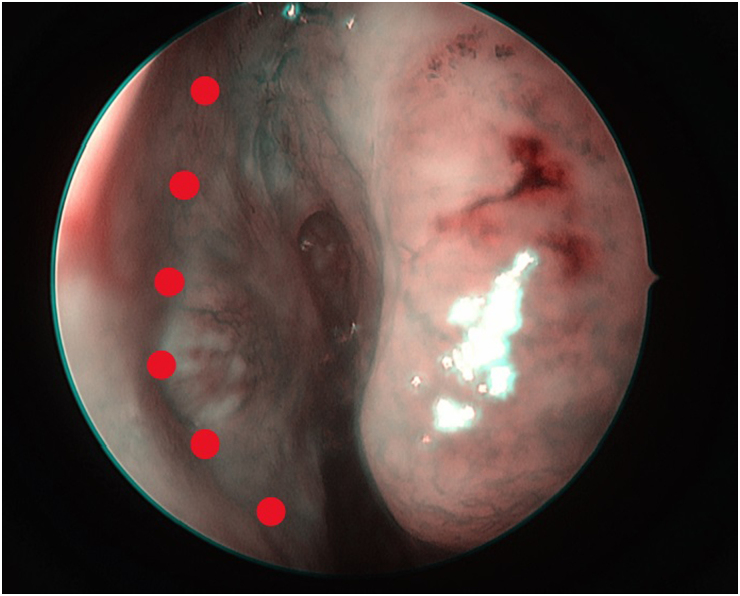
Endoscopic evaluation of the left nasal cavity. Narrow-band imaging shows increased vascularity, at the area where the tumour is involving the superior nasal septum (red dots).

**Figure 2:**
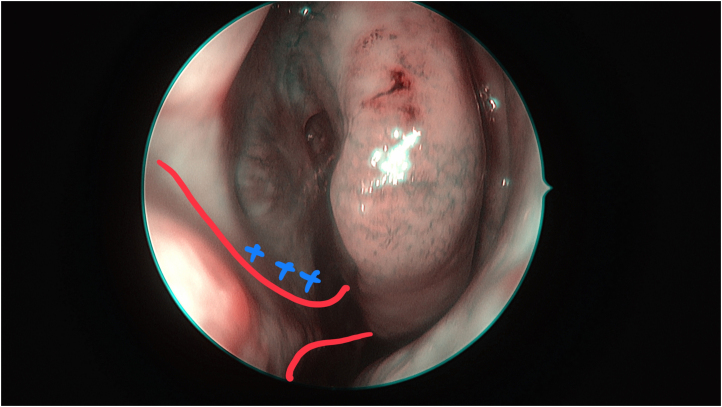
A biopsy for the frozen section was taken from the superior edge of the proposed flap (blue crosses) and the nasoseptal flap was designed as shown (red line). Because of the location of the pathology, the flap was extended to the nasal floor up to the level of the inferior meatus.

In cases in which the tumour involves the superior septum, careful planning of the flap and clear edges are of paramount importance (Figure 1). Our standard practice is to obtain tissue samples from macroscopically clear areas for frozen sections. This is an essential step to ensure avoidance of tumour seeding at the area of reconstruction. Following narrow-band imaging (NBI) mapping of the septum and nasal cavity, we take a biopsy from the superior edge of the proposed flap (Figure 2) for frozen section. Dual confirmation of the tumour-free edge helps us to proceed with safe reconstruction. The flap is raised following the standard technique, extending to the nasal floor up to the inferior meatus, ensuring Hasner's valve remained intact. On follow-up scans of our cases, there was no evidence of any residual disease related to the flap.

The principle behind NBI lies in the use of specific narrow-bandwidth filters that illuminate tissue with light at wavelengths corresponding to the peak absorption of haemoglobin.^1^ More specifically, NBI utilises narrow-band spectrum optical filters to emit light at two specific wavelengths, 415nm (blue) and 540nm (green).^2^ By highlighting vascular structures and mucosal patterns, NBI facilitates the identification of lesions, inflammatory changes and neoplastic growths with remarkable precision. One of the notable advantages of NBI is its ability to differentiate between benign and malignant lesions.^3^ NBI technology can represent a useful adjunct in the planning of anterior skull base reconstruction, assisting towards optimal flap viability and tumour-free edges. Further studies and larger study samples are required to draw safe conclusions about this technique.
